# The Royal Devon and Exeter Hospital

**Published:** 1915-05-08

**Authors:** 


### THE DEPARTMENT OF MODERN NURSING.*

### XII.—The Royal Devon and Exeter Hospital.

The Royal Devon and Exeter Hospital contains 200 beds, for the occupants of which there is a nursing staff of about sixty-two. In case of necessity, additional nurses are brought in from the staff of private nurses, of whom there are generally between thirty and forty. The matron believes in promoting her own trained probationers to be sisters and staff nurses, and during the last fourteen years only one sister trained elsewhere has been added to their number. The matron herself also received her training within the same walls.

The length of service required to gain a certificate is four years; the first three being spent entirely in the hospital, and the fourth in private nursing. Candidates must be between the ages of twenty-one and thirty-five; the matron, however, strongly prefers the former age. She relates that for one year she tried the experiment of taking only probationers over twenty-two, but found at the end of their probation that they did not seem superior in any way to those who had begun at twenty-one. The agreement is signed after a trial of three months, during which time three reports from the matron and house surgeon are presented to the house committee, and a member of the honorary staff reports on the candidate's physical fitness for the work. In consequence of this careful scrutiny, few probationers are weeded out after the first three months, and although they are liable to dismissal for neglect of duty or other faults, they are first entitled to an interview with the committee.

A fair education is required, and this is not held to include merely an elementary school course. Three courses of lectures are given to the probationers in the first two years, and they must pass examinations in them before being promoted to be staff nurses. The first course consists of eight lectures by the matron on general nursing, and seven by the house surgeon on various subjects in connection with it, asepsis, reporting cases, etc. The next course is one of fifteen lectures given by a member of the medical staff on medical nursing; and the third fifteen given by one of the surgeons, on surgical nursing. In addition there are lectures by a member of the staff on ophthalmic nursing, and by the sisters on massage and electrical treatment.

Six cookery lectures are given every year by a qualified teacher. It is not compulsory for every nurse to take the massage examination, but each one must learn enough practical massage from the qualified massage sister to be able to do what is required in the wards. Apart from the lack of opportunity to learn maternity work or dispensing in the hospital, there is every variety of work. Each nurse has a turn in the children's ward and a month in the theatre, and though there is no gynæcological ward, there are many cases in the women's wards which supply material for training the nurses. Not all the nurses can go through the electrical department, but all may attend the lectures, and may spend occasional afternoons when off duty in watching the electrical work. Perhaps this is a vestige of the former custom in this hospital of sending the probationers to sit in the theatre and watch the operations, a custom now happily abolished. (See The Hospital, November 6, 1909, p. 160.)

Gold and silver medals are awarded every year to the successful candidates in the examination, and marks given by the sisters in their reports are added to the marks recorded by the examiner, so that the medals represent success both in theoretical and practical nursing.

The off-duty time is on a liberal scale, partly owing to the relaxing climate of Exeter, a climate in which a woman could not continually do such hard work as she might be expected to do in London. Nurses are off duty two and a-half hours daily, and every third Sunday have seven and a-half hours off, with one day a month from 10 a.m. to 9 p.m. It will be observed that this does not allow the precious privilege of breakfast in bed occasionally. The time on duty works out at nine and a half hours a day. Twenty-one days' holiday are allowed in the year.

Probationers have no salary for the first six months. For the next six months they are paid £5, and for the second and third years at the rate of £20 a year. In the fourth year, a nurse placed on the private staff is paid £25; she receives £35 for the next year, and then an additional £5 for each year is given till the maximum of £50 is reached. If in the fourth year she is placed on the hospital staff as nurse, she is paid £25, while the sisters' salary rises from £40 to £50 by £2 10s. yearly.

There is no nurses' home in Exeter Hospital. Some of the nurses are lodged in two small houses, one containing twenty-six and the other eighteen beds, and the remainder sleep in rooms leading off each side of a very dark passage contrived in the oldest part of the hospital out of a disused ward. The matron hopes that by the end of a year or two this very inconvenient and objectionable arrangement will be replaced by an additional wing to be built on to the larger of the two houses now occupied. When that is accomplished, no doubt a home sister will be added to the staff; at present the matron has the entire charge of the nurses, including both hospital and private staff.

At the end of the training most of the nurses take up private work, though some seek for institution appointments. Many of them, of course, are needed to keep up the hospital staff, which is always fluctuating on account of losses. No fewer than seven sisters were needed in nine months last year!

The hospital does not offer any advantages to its nurses and sisters who wish to join the Royal National Pension Fund. This hospital needs modernising structurally and otherwise.

* Previous articles: May 16, 30; June 13; July 11, 25; Aug. 8, 1914; Jan. 16, 30; Feb. 27; March 13; April 3, 1915.

